# Steppingstones to the implementation of an inhospital fracture and dislocation registry using the AO/OTA classification: compliance, completeness and commitment

**DOI:** 10.1186/1757-7241-18-54

**Published:** 2010-10-18

**Authors:** Terje Meling, Knut Harboe, Astvaldur J Arthursson, Kjetil Søreide

**Affiliations:** 1 Department of Orthopaedic Surgery, Stavanger University Hospital, Stavanger, Norway; 2 Department of Surgery, Stavanger University Hospital, Stavanger, Norway; 3 Department of Surgical Sciences, University of Bergen, Bergen, Norway

## Abstract

**Background:**

Musculoskeletal trauma represents a considerable global health burden, however reliable population-based incidence data are scarce. A fracture and dislocation registry was established within a well-defined population. An audit of the establishment process, feasibility of the registry work and report of the collected data is given.

**Methods:**

Demographic data, fracture type and location, mode of treatment, and the reasons for the secondary procedures were collected and scored using recognized systems, such as the AO/OTA classification and the Gustilo-Anderson classification for open fractures. The reporting was done in the operation planning program by the involved orthopaedic surgeon. Both inpatient and day-case procedures were collected. Data were collected prospectively from 2006 until 2010. Compliance among the surgeons and completeness and accuracy of the data was continuously assured by an orthopaedic surgeon.

**Results:**

During the study period, 39 orthopaedic surgeons were involved in the recording of a total of 8,188 procedures, consisting of primary treatment of 4,986 long bone fractures, 467 non long bone fractures, 123 dislocations and 2,612 secondary treatments. In the study period 532 fractures or dislocations were treated at least once for one or more serious complications. For the index year of 2009, a total of 5710 fractures or dislocations were treated in the emergency department or hospitalized, of which the 1594 (28%) were treated at the inpatient or day-case operation rooms, thus registered in the FDR. Quality assurance, educational incentives and continuous feedback between coders and controller in the integrated electronic system are available and used through the features of the electronic database.

**Conclusions:**

Implementing an integrated registry of fractures and dislocations with the electronic hospital system has been possible despite several users involved. The electronic system and the data controller provide for completeness and validity. The FDR has become an indispensable tool for the department for planning and education and will serve as a prerequisite for the conduct and execution of future prospective trials within the department. Further, other departments with similar electronic patient files may fairly easily adopt this system for implementation.

## Background

Major trauma is a leading cause of death and disability around the world[1]. In Scandinavia, the incidence of severe injury ranges from 30 to 52 per 100,000 inhabitants annually, with about 90% due to blunt trauma[2]. Notably, musculoskeletal injuries are very common and represent a considerable global health burden, and long bone fractures take up five of the top ten most frequent non-fatal injuries sustained after trauma worldwide[3]. Fractures alone account for significant morbidity during childhood as well as in the elderly, albeit different fracture patterns are known to occur [4-6]. Although data exist on musculoskeletal injury epidemiology, discrepancy in the aim and focus of most studies hampers drawing firm conclusions. Further, it is often difficult to find common datasets and definitions when comparing national statistics of injury [7-10]. Thus, the literature is somewhat conflicting in the incidence reports of fractures as definitions, access to data, and diagnostic systems differ widely [6,11-15].

Consequently, heterogeneity is widely present among the studies, and, drawing firm conclusion from any one study is thus hampered. To the best of our knowledge there is a paucity of prospective, population-based studies investigating the incidence patterns of age- and sex-specific patterns of long bone fractures requiring inpatient or day-case procedures. This contrasts the WHO call for increased focus on these injuries, both in high-income and low-income countries [16].

The aim of this study is to audit the experience and present the steppingstones to the implementation and establishment of an inhospital-based registry of all fractures and dislocations requiring inpatient or day-case treatment in a Norwegian trauma centre using the recognized orthopaedic fracture AO/OTA classification system.

## Methods

### Study population

Stavanger University Hospital (SUH) serves as the only primary trauma and emergency care facility for a mixed population of about 317,000 inhabitants (2008 statistics) [17] in the South-Western part of Norway. The SUH orthopaedic department, in principle, covers all aspects of general orthopaedic trauma and non-trauma orthopaedic surgery for all age groups, with some exceptions (e.g. complex fractures of the pelvic ring and the acetabulum, fractures and dislocations of the face, head, neck, complex fractures of the hand and some of the fractures of the thoracic and lumbar spine).

The primary catchment area has an urban: rural ratio of about 5:1 [17]. While having a growing population, the SUH serves as the only primary health care facility for the population under investigation and, thus, providing for reliable incidence and epidemiological investigations of disease in this region over time. Consequently, the study population should be well representative of other Western, non-metropolitan regions.

### Inclusion and exclusion criteria

Included in the FDR are all procedures performed for fractures or dislocations, which required inpatient or day-case procedures at the operation rooms by the orthopaedic department at SUH. Secondary procedures (reoperations and revision surgery) are also included. Current study period was June 19^th^, 2006 until March 10^th^, 2010.

Treatments performed in the emergency department are not included, e.g. fractures solely given plaster cast, with or without reduction. As closed reduction and plaster casting of children are most often done in general anaesthesia, these procedures are included in the FDR. Procedures to patients living outside the region, but treated at SUH are included. Fractures operated in other departments than the orthopaedic department (e.g. plastic & reconstructive surgery; neurosurgery) at SUH are not included. Patients of all ages are included.

### Data collection and database

After a two and a half year period of running a manual registry (data not reported), the digital registration into the electronic FDR started, and as such represents the current period of this study, starting as of June 19th, 2006.

**Figure 1:**
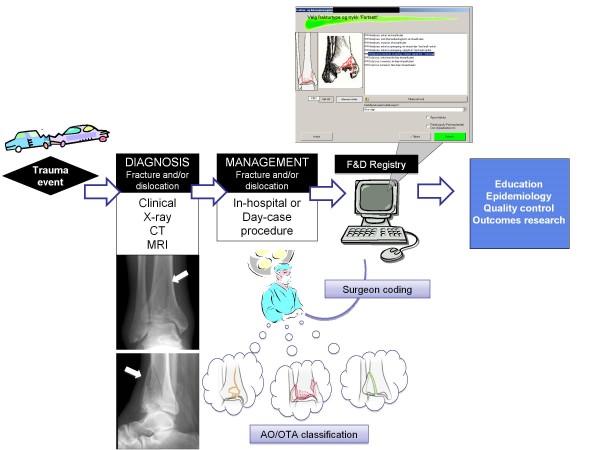
**Flow chart of Registry use**. Flow chart of the use of the FDR in the orthopaedic department.

The FDR Board decided the variables included in the FDR. The Board also approved the FDR protocol, which includes the history, the aim, the definitions, and the variables of the FDR. Any given change will be listed in the protocol. Ahead of the digital introduction the surgeons went through a teaching program which included digital assignments. All orthopaedic operations are consecutively registered by the orthopaedic surgeon in charge of treatment (figure 1) into the operation planning program ORPlan. The ORPlan programme includes a fracture and dislocation module where all the data to the FDR are registered. Both primary and secondary procedures, along with the reasons for the secondary procedures, are registered.

To ensure the quality, the parameters are recorded as alternatives in scroll-down menus. There is an extensive use of tool tips, containing definitions and explanations. The fracture classification coding is done in a stepwise manner. First the fractured bone is chosen (e.g. femur), then the bone segment (e.g. shaft). Finally the correct AO/OTA-type and AO/OTA-group is chosen by selecting the most correct definition for each different AO/OTA-group. Simultaneously the illustration of each chosen AO/OTA-group is shown (figure 1). Depending on the AO/OTA code and the fixation devices noted ORPlan suggests the diagnose (ICD-10) and the procedure (NCSP).

Any given operational event may have one or more procedures (for instance bilateral fracture treatment of the distal radius) and, accordingly, there is a registered procedure for every fracture or dislocation. The ORPlan includes fracture registry reports available for all surgeons involved to check for content. Furthermore, more advanced reports are available for the controller for extracting data.

Anaesthesiologists, nurses and surgeons all use the program for planning, scheduling and registration of all operative procedures. The program ensures complete coverage of all patients admitted for surgery or treatment under general anaesthesia, as it is only possible to operate when a patient is scheduled in the program. Features of the FDR are given in table 1.

**Table 1:** Features of the FDR

Feature	Comment
**Feasibility and** **systems integration**	Integration with daily practice and OR planning Well-tailored software for integration Integration with electronic hospital files Integrated with coding records (diagnosis and procedures; e.g. ICD-10 and NCSP/NCMP)

**Compliance**	Integrated with planning and registration of all operative procedures User friendly working tool among surgeons Step-by-step approach for completing data Use of recognized scoring system (AO/OTA) and Gustilo-Anderson (open fractures)

**Education**	Integration of AO/OTA coding with illustrated definitions and descriptions Feedback by controller Data for internal review and education

**Quality assurance**	Data controlled, corrected and completed by an orthopaedic surgeon Allows regularly or periodic overview of procedures and complications in department Allows for data extraction at individual/surgeon, procedure or fracture/dislocation, or/and department level Allows for prospective trials and studies.

### Description of the database working key

ORPlan is an operation planning program which includes a fracture and dislocation registering module. The system is currently based on a SQL Server (Microsoft(tm)) and thin clients made in VB.net.

### Classification

The Registry is based on the Müller-classification of long bone fractures [18] and the Orthopaedic Trauma Association's (OTA's) Fracture and dislocation classification compendium - 2007[19]. Fractures are scored based on the best knowledge of the surgeon obtained at the end of the operation as informed by both radiological as well as surgical exposure. In principal, intraoperative findings, and preoperative X-ray plain films are almost always included, but often also computed tomography with or without three-dimensional (3D)-reconstructions or magnetic resonance imaging in a select manner.

### Data control

A control of the registered parameters and completion of data is continuously performed by one orthopaedic surgeon (Terje Meling). The process follows several steps to ensure completeness and validity. For one, the ORPlan is monthly searched for non-coded orthopaedic operational events. Second, the operational protocol procedures, not reported as fractures or dislocations, are monthly searched for procedures which still should be reported to the FDR. Third, most of the patients' operation-notes are briefly searched for completion and quality assurance of the parameters. Close attention is being paid to the following:

a) The registration of whether the fracture event previously has been treated at SUH (outpatient or inpatient), or at another hospital (i.e. reason for inclusion or exclusion);

b) If the fixation method used is correctly reported;

c) If any secondary procedures are not correctly linked to the primary procedure registration;

d) If the indications for the secondary procedures are correctly reported, and;

e) If the open fractures and pathological fractures are correctly reported. Finally, most of the patients x-rays are reviewed to ensure the AO/OTA fracture and dislocation classification and to ensure that simultaneous dislocations are registered, if present.

ORPlan includes a feedback reporting system. Thus written feedback is given (from the controller) to the coding surgeons when important failures or discrepancies are found in the registrations. When the surgeon enters the ORPlan for the next time the feedback report pops up on the screen.

### Definitions

#### Fractures

Fractures are defined as a single or multifragmentary circumferential disruption of a diaphysis or metaphysis or a single disruption of an articular surface [18,20]. In the immature skeleton the disruption might not be complete. Hence bowing, torus and greenstick fractures are classified as fractures in the Fracture and dislocation classification compendium for children [21]. Each of the long bones were, according to Müller, [18] divided into the proximal, diaphyseal, or distal segment. Only one fracture was registered in one bone segment at any one occasion. Usually one fracture is counted even if more than one segment of a bone is fractured, depending on the fracture pattern. The long bones are classified into fracture group, using four signs of the AO/OTA classification. The non long bone fractures are only classified into fracture segment (the two first signs of the classification). Open fractures are scored according to the modified Gustilo-Anderson classification [22,23].

#### Dislocations

Dislocations are defined as displacement of the main part of one side of the joint which needs immediate reduction. Or when the main intraarticular surface is or has been dislocated more than one third of the width of the joint. The dislocation is described by the AO/OTA segments proximal and distal to the joint. For instance an elbow dislocation is called 13-21, and a dislocation of the syndesmotic joint in the ankle is called 43-43. Fractures with dislocation of the joint are classified both as fractures and dislocations.

#### Primary and secondary procedures

As each fracture in any patient may be operated on at several times, any first procedure (e.g. a fixation of a femoral neck fracture with collum screws) is called the primary procedure. Any subsequent procedure (e.g. need for removal of the screws and subsequent hemiarthroplasty) is thus referred to as a secondary procedure. In addition, all secondary procedures are linked to the original, or index treatment of the fracture, so as to allow complete tracking of all subsequent procedures for any given injury in a patient. Each injury might have many secondary procedures. Indications for the secondary procedures are registered accordingly. Each secondary procedure might be performed for more than one indication/reason. Consequently, the secondary procedures and the indication (e.g. planned or, required from primary treatment failure) constitute the quality measure of the fracture and dislocation treatment in the department. Some of the reasons are more serious than others and represent the severe complications (table 2). Only complications that involve a procedure at the operation are registered.

**Table 2:** Indications for performing secondary procedures either by intention (planned, scheduled) or, based on failed primary (index) management

Indications for secondary procedures (reoperations)		
**Severe complications or major events**	1	Wound disruption
	
	2	Deep infection
	
	3	Deep hematoma
	
	4	Refracture (inadequate trauma)
	
	5	Peri-implant fracture
	
	6	Osteonecrosis
	
	7	Delayed/non union
	
	8	Malunion
	
	9	Posttraumatic arthritis
	
	10	Implant broken through joint surface
	
	11	Implant failure
	
	12	Loosening of arthroplasty material
	
	13	Dislocation after hemiarthroplasty
	
	14	Dislocation after total joint replacement
	
	15	Debridement (secondary soft tissue damage)
	
	16	Other (serious reasons)

**Mild complications or minor events**	17	Failed index conservative procedure
	
	18	Superficial infection
	
	19	Superficial hematoma
	
	20	Refracture (adequate trauma)
	
	21	Discomfort from osteosynthetic material
	
	22	Other (inconvenient reasons)

**Planned/intended events**	23	Debridement (primary soft tissue damage)
	
	24	Planned final fixation
	
	25	Routine metal removal
	
	26	Other (intended reasons)

Calculated are, 1) the numbers of secondary procedures, 2) the numbers of indications and 3) the numbers of injuries (fractures or dislocations) requiring secondary procedures. For instance a hip fracture primary treated with hip screws required three secondary procedures: a) Replacement by total hip-arthroplasty because of metal failure, secondary arthritis and non-union. b) and c) Two wound debridement due to deep infection. 1) Three secondary procedures, 2) four serious indications, and 3) one fracture are measured.

### Study ethics

The registry is approved by the Norwegian Social Science Data Service. Consent for this quality assurance project is given by the Regional Ethics Committee.

### Statistical analysis

The data reported herein are descriptive and no statistical comparison has been performed.

## Results

During the study period 8,188 procedures were recorded, consisting of primary treatment of 4,986 long bone fractures, 467 non long bone fractures, 123 dislocations (without fracture) and 2,612 secondary treatments. In the same period 532 fractures or dislocations were treated at least once for one or more severe complications (defined in table 2). The incidence patterns for primary procedures of long bone fractures (all treated at the SUH) in this population has been described in detail elsewhere [4]. Baseline characteristics in the current study are given in table 3.

**Table 3:** Baseline characteristics of data in the FDR

	Sex		Type of treatment		Type of injury		
	**Female** N (%)	**Male** N (%)	**Primary** **†** N (%)	**Secondary** **‡** N (%)	LBF * N (%)	NLBF ** N (%)	Dislocation *** N (%)

**adult**	3793 (55)	3128 (45)	4666 (67)	2255 (33)	6053 (88)	708 (10)	160 (2)

**child**	501 (40)	766 (60)	910 (72)	357 (28)	1184 (93)	58 (5)	25 (2)

**† **The first time a fracture was treated at the operation

**‡ **Subsequent treatments

* Fracture with or without dislocation in long bones; humerus, radius, ulna, femur, tibia or fibula

** Fractures with or without dislocation in other bones (non long bone fractures).

*** Dislocations without any significant fracture

### Adult population (≥ 16 years of age)

In the adult age group, women constituted 59% of the primary treatments and 47% of the secondary treatments. For the primary treatments the median age for women was 73 years, and for men 49 years of age. The most frequently fractured long bone segments were the proximal femur (40%), the distal forearm (22%) and the ankle (16%). Secondary procedures for the long bone fractures were most often recorded for fractures in the ankle (27%) and in the proximal femur (23%).

### Children (< 16 years of age)

Girls made up a total of 40% of the primary treatments and 38% of the secondary treatments. For the primary treatments the median age among the children was 9 years for girls and 10 years for boys. The most frequently fractured long bone segments among the children were the distal forearm (38%), the diaphyseal forearm (25%) and the distal humerus (15%). Secondary procedures for the long bone fractures were most often executed in the shaft of the forearm (29%) and in the distal humerus (27%).

### Non long bone fractures and dislocations

The most common primary treated non long bone fractures and dislocations are presented in table 4.

**Table 4:** Fractures and dislocations (counted at their primary treatment)

Bone site (segment)	Code †	N	%	Bone site (segment)	Code†	N	%	Joint	Code‡	N	%
Prox Femur	31	1653	33	Metacarpal	77	99	21,2	Acromeoclavicular	15-14	31	25

Distal forearm	23	1211	24	Clavicle	15	79	16,9	Elbow	13-21	28	23

Ankle	44	682	14	Hindfoot	*	65	13,9	Hip	62-31	14	11

Forearm diaphyseal	22	267	5	Phalangeal (Hand)	78	62	13,3	Humeroscapular	14-11	13	11

Femoral shaft	32	198	4	Metatarsal/phalangeal (Foot)	87/ 88	62	13,3	Lis Franc	85-87	9	7

Prox forearm	21	173	4	Patella	34	43	9,2	Syndesmotic	43-43	7	6

Distal humerus	13	168	3	Pelvic ring	61	16	3,4	Metatarsal or phalangeal	**	4	3

Distal tibia	43	164	3	Lumbar spine	53	13	2,8	Distal radioulnar	23-23	2	2

Tibial shaft	42	155	3	Other		28	6,0	Other		15	12

Proksimal tibia	41	112	2								

Proximal humerus	11	107	2								

Distal femur	33	54	1								

Humeral shaft	12	42	1								

Total		4986	100	Total		467	100	Total		123	100

**† **OTA classification code; bone segment (version 2007)

**‡ **Proximal and distal bone segment (OTA 2007) involved in the dislocation

* OTA classification code (2007): 81, 82, 83, 84 and 85

** 87-87, 87-88 or 88-88

### Day-case surgery

Fractures treated primarily as day-case surgery increased from 2007 to 2009, (data not shown). However, the overall numbers were small compared to the total in-hospital management. The overall number of primary treatments increased during the study period, whereas the secondary treatments did not. Notably, almost one-third of all secondary treatments where performed as day-case procedures (mostly uncomplicated hardware removals).

### Emergency unit

For the index year of 2009, a total of 5710 fractures or dislocations were treated in the emergency department or hospitalized, of which 1594 (28%) were treated at the inpatient or day-case operation rooms and registered in the FDR.

### The study region

If the patient acquires a fracture when travelling outside the region, the primary treatment will be performed at another hospital and, consequently, not recorded in the FDR. The Statistics Norway reported, for the index year of 2006, the numbers of inhospital patients living in our region treated for long bone fractures (ICD-10 codes; S42, S52, S72 and S82). The data were divided into those treated at SUH and those treated elsewhere. Seven per cent of the patients had their primary treatment at another hospital. Simultaneously, five per cent of the primary treated fractures, recorded in the FDR, had their residency located outside the region.

### Feasibility and compliance

During the study period all the 39 orthopaedic surgeons involved with fracture treatment recorded a mean of 208 (range 2-652) procedures to the Registry. The 39 surgeons were all department surgeons in charge in the majority (99%) of the procedures. Guest-surgeons, trainees or surgeons from other surgical specialties at SUH were in charge of the remaining 1% of the registered procedures, thus recorded by the orthopaedic assistant.

Assuming optimal soft- and hard-ware conditions, the four-stage validation and completion process (, as described above,) takes about 1-2 days work (12-15 hours) per month. There has not been a complete measurement of the exact number of missing or misclassified registrations that has eventually been completed or corrected during the study period. However, as a proxy for evaluation of the above described validation process, the following has been done:

For the first and second step one month was arbitrarily selected (August 9^th ^until September 8^th ^2010):

First step: Nine out of 371 (2%) orthopaedic procedures were not coded. Of these one procedure was due to fracture (or dislocation) and should have been included in the FDR.

Second step: A total of 11 out of 211 (5%) procedures were misclassified as non-fracture/dislocation procedures thus had to be recoded into the FDR. All of the registrations misclassified were secondary procedures. Seven of those were repeated wound debridement either for *deep infection *or *soft tissue damage*, and two were *routine metal removals*.

For the third step the arbitrarily chosen index months October and November 2009 were evaluated for correctness as mentioned in a-e:

a) Four out of 346 (1%) procedures were not related to any fracture or dislocation, consequently had to be removed from the FDR.

b) Four out of 127 (3%) secondary procedures failed to be marked in the check box: *Former inpatient procedure at SUH*. Two out of 8 (13%) failed in the check box: *Former treated at another hospital.*

c) Two out of 346 (0,6%) of the registrations of the *Main fixation method *were corrected.

d) Three out of 127 (2%) secondary procedures were not correctly linked to their index treatment.

e) Ten out of 127 (8%) secondary procedure registrations were corrected for one or more of the indications considered serious (table 2).

For the fourth step the arbitrarily year of 2008 were chosen. The controller changed the AO/OTA four-sign-code in 312 out of 1319 (24%) of the long bone fractures, in two out of 118 (2%) of the two-sign-code for the non long bone fractures and in one out of 27 (4%) dislocations. 140 out of 186 (75%) fractures with simultaneous dislocation missed the dislocation code.

During the last year of the study period, a total of 120 feedbacks were written to the surgeons.

## Discussion

This study describes important steppingstones to the implementation and use of a fracture and dislocation registry in a busy orthopaedic department. We believe the compliance among the involved number of surgeons have been satisfactorily high throughout the study period and in large parts driven by the electronically integrated database system, which allows for both scheduling of surgery as well as coding of the named injuries. Completeness has been assured with only minor deviations and need for corrections by the controller. The latter has been instrumental in maintaining the registry and, as such, emphasizes the commitment needed to run a quality assurance database in routine, clinical practice.

Most studies regarding the incidence of fractures consider both inpatient and outpatient treatments[6,24]. The frequent bone bruises, the compression fractures of the vertebras and the undisplaced or even incomplete fissures to the nose, ribs, fingers and toes are not considered in the current study. Rather, the indication for surgery is a crucial criterion for the recording rate to the FDR. Of the inpatient and outpatient fractures or dislocations that occurred in the catchments area of SUH (for the index year of 2009) some 28% were treated in the inpatient or day-case operation rooms. This finding is consistent with the hospitalized number of patients (about one third) reported from Trondheim, Norway[24].

Some limitations to the current registry need to be mentioned. For one, the injury mechanism is not registered in the FDR. The surgeon does not always have the exact knowledge, thus the timing of the registration for this issue is not optimal. However, the purpose of the FDR is more related to the quality of the fracture treatment than to the prevention of traumas. However, this aspect needs further consideration in the future.

Second, dislocations are, while not being fractures, also closely related to the skeleton. Dislocations are not as frequent as fractures, but undoubtedly clinically important and may be disabling for the patient. Instead of excluding the dislocations we have included them as a special entity in the current registry. The Fracture and dislocation classification compendium of OTA [19] includes a special classification for dislocations. The compendium does not include the definition of a dislocation. The classification of the joint involved is not very desciptive. Consequently, the dislocations may be better descibed by the proximal and distal AO-segment involved (e.g. tibiofemoral dislocations were recorded as 33-41 and patellofemoral as 33-45). Often the dislocations have a subsequent bony avulsion, in the case of which the fracture-dislocation will receive both a fracture- and a dislocation-code. However, likely based on the somewhat unclear definition and recording format, the registration of the simultaneous dislocation in fracture-dislocations was the most frequently missing coding. Also, secondary procedures and especially their indications were more often missed or misreported, before correction. As such, "problem areas" have been identified in the coding practice; these findings have imposed some structural changes in the registering format in ORPlan. We believe the structural steps for quality assurance as explained above have ensured a high completeness rate in the registry.

Third, while being the most widely used classification system for orthopaedic trauma internal validity of the AO/OTA-classification has been questioned [19]. We believe the use of scroll-down menus with illustrations and definitions used in the FDR, the timing of the coding in relation to admission and treatment by all orthopaedic surgeons and the continuous control ensure reasonable validity. However, to substantiate this view a more extensive project investigating coding validity and reliability is being undertaken and will be presented separately.

Hospital registries serve many purposes and might be beneficial for the patient, physicians and administrators [25]. National registries like the National Hip Fracture Registry in Norway have immediate advantages concerning the large numbers [26]. Both the primary and secondary treatments are considered even if procedure is performed at other hospitals in the country. Notably, the more uniform diagnosis and management in a single hospital makes the data easier to present and compare. The development and implementation of a registering soft-ware that can be inter-linked with each hospital's electronic files may be difficult to perform. As is the experience in this project, it would be necessary to have a committed controller [25] to ensure high compliance and completeness.

The FDR has become a natural part of the departmental routines for scheduling, planning and evaluation of fracture and dislocation management in our department. Further, it is used as educational feedback and quality assurance tool at both individual and departmental levels.

## Conclusions

Implementing an integrated registry of fractures and dislocations with the electronic hospital system has been possible despite several users involved. The electronic system and the data controller provide for compliance and completeness. The system contains available registry reports for the surgeons. The FDR has become an indispensable tool for the department for planning and education and will serve as a prerequisite for the conduct and execution of future prospective trials within the department. Further, other departments with similar electronic patient files could fairly easily adopt this system for implementation.

## Abbreviations

AO: Arbeitsgemeinschaft für Osteosynthesefragen; FDR: Fracture and Dislocation Registry at Stavanger University Hospital; ICD-10: International Classification of Diseases; NCSP: The Nordic Medico-Statistical Committee Classification of Surgical Procedures; NOMESCO: Nordic Medico-Statistical Committee; ORPlan: Operation Room Planning Program; OTA: Othopaedic Trauma Association; SUH: Stavanger University Hospital.

## Competing interests

The authors declare that they have no competing interests.

## Authors' contributions

TM is the supervisor of the registry, carried out control of completeness and accuracy of the register and contributed to data extraction and writing. KH made the operation planning program; ORPlan and contributed to the data extraction. AJA contributed in the planning and participated in the writing process. KS greatly participated in the design and writing of the study. All authors read and approved the final manuscript.
